# Effects of High-Acuity Cases on Pediatric Emergency Department Flow: A Combined Quantitative and Qualitative Analysis

**DOI:** 10.7759/cureus.112046

**Published:** 2026-07-04

**Authors:** Casey M Jones, Melanie Doyle, Brett Taylor, Michael Young, Jason Emsley, Ronald May

**Affiliations:** 1 Department of Emergency Medicine, Dalhousie University, Halifax, CAN; 2 Department of Emergency Medicine, IWK Health Centre, Halifax, CAN; 3 Department of Emergency Medicine, QEII Health Sciences Centre, Halifax, CAN

**Keywords:** emergency department efficiency, emergency department flow, pediatric emergency department, resuscitation, trauma team activation

## Abstract

Introduction

The impact of trauma team activations (TTAs) and other high-acuity cases on pediatric emergency department (PED) flow is not well understood. We sought to compare the operational impact of high-acuity cases on PED flow, both from the provider perspective and through analysis of patient visit data.

We sought to elucidate the effects of trauma and other high-acuity cases on emergency department flow (and disruption), using a combined quantitative and qualitative approach.

Methods

Visit data were analyzed for patient time-to-doctor during TTAs and non-TTA CTAS-1 (Canadian Triage and Acuity Scale) events between 2009 and 2019 at a single tertiary care trauma center. Time-matched control periods were similarly evaluated. A questionnaire was distributed to a multidisciplinary staff group at the same center, including 12 Likert-scale and five optional free-text questions. Quantitative results were compared using chi-square analyses, while grounded theory thematic analysis was applied to analyze free-text responses.

Results

Of 1305 eligible visits analyzed, TTAs did not significantly increase time-to-doctor. Non-TTA CTAS-1 visits (such as resuscitations due to sepsis) resulted in significantly longer time-to-doctor compared to TTAs and controls (p<0.001). Left without being seen rates were similar across groups. Among 51 respondents, 88% and 90% reported that TTAs and non-TTA CTAS-1 events, respectively, disrupt department flow. Nursing staff were significantly more likely than physicians to perceive a negative impact of non-TTA CTAS-1 cases on patient safety and clinical care (80% vs. 30%, p=0.012). Themes from free-text comments included staff shortages, care disruptions, resource strain, and quality improvement needs.

Conclusion

Non-trauma resuscitation ED visits may be more disruptive to department flow compared to TTAs. Aligning frontline staff perceptions with objective data will be critical for developing quality improvement strategies to optimize efficiency during high-acuity events.

## Introduction

In children, injury is the leading cause of preventable death in Canada and the United States [1,2]. A key component of caring for trauma patients is the use of a specialized, multidisciplinary trauma team response. Strong evidence demonstrates that patients benefit from specialized trauma care [3-5], which is associated with decreased time to diagnostic imaging and definitive management [6,7].

Trauma care centers use defined criteria to call a trauma team activation (TTA) that assembles the on-call trauma team to respond in the emergency department for patient care. While there are subtle variations in activation criteria from center to center, all of these include physiologic, anatomic, and mechanistic factors. The trauma team responding to a TTA consists of healthcare professionals from varied backgrounds, including emergency medicine, surgical specialties, anesthesia, nursing, respiratory therapy, and others [8].

All patients presenting to Canadian emergency departments are triaged using the Canadian Triage and Acuity scale (CTAS) [9]. A similar scale exists for pediatric patients (PaedCTAS), which has a physiological approach and uses symptomatology to assign a triage level [10]. The CTAS scale consists of five triage levels, each corresponding to clinical presentation severity. Pediatric EDs in Canada utilize the PaedCTAS to identify patients with higher acuity needs and to help ensure they are seen by a physician in a timely manner.

High-acuity cases like trauma and other emergent resuscitations require a significant amount of departmental and staff resources. A 2009 systematic review identified that overcrowding affects mortality outcomes, patients leaving without being seen, time to treatment, and longer length of stay [11]. A 2019 multi-center Canadian pediatric emergency department (PED) study showed that overcrowding was not associated with hospital admission within seven days of ED visit but was associated with increased return visits among low-acuity cases [12].

Little is known about the impact that TTAs and other high-acuity cases have on PED flow. We performed a detailed quantitative and qualitative analysis to understand the effects of high-acuity cases on flow and crowding in a PED from the provider's perspective. We also evaluated the effect of TTAs and other CTAS-1 patients on patient flow. The primary objective of this study was to compare the operational impact of TTA and non-TTA CTAS-1 resuscitation events on PED flow. Specifically, we aimed to measure flow using two objective visit metrics: patient time-to-doctor and left without being seen (LWBS) rates between TTAs and non-TTA CTAS-1 cases. Secondarily, we sought to evaluate provider perceptions of the disruption to patient safety and clinical care associated with high-acuity cases.

## Materials and methods

Visit analysis

Emergency visit data from 2009 to 2019 were analyzed to select CTAS 1 visits. A level-1 CTAS patient is considered a resuscitation patient and should be seen immediately. Example presentations include cardiorespiratory arrest, severe respiratory distress, and severe shock. A TTA was also considered a CTAS level-1 case. CTAS-3 cases are considered urgent (e.g., abdominal pain), and CTAS-4 and -5 cases are less urgent and non-urgent, respectively. Level-1 CTAS visits were filtered to be at least 12 hours from any previous CTAS-1 visit, and at least six hours from any subsequent CTAS-1 visit. These timeframes were selected pragmatically based on our department throughput, and to avoid confounding the data with the impact of multiple CTAS-1 events. These visits were labeled “unconflicted” CTAS 1 events.

**Figure 1:**
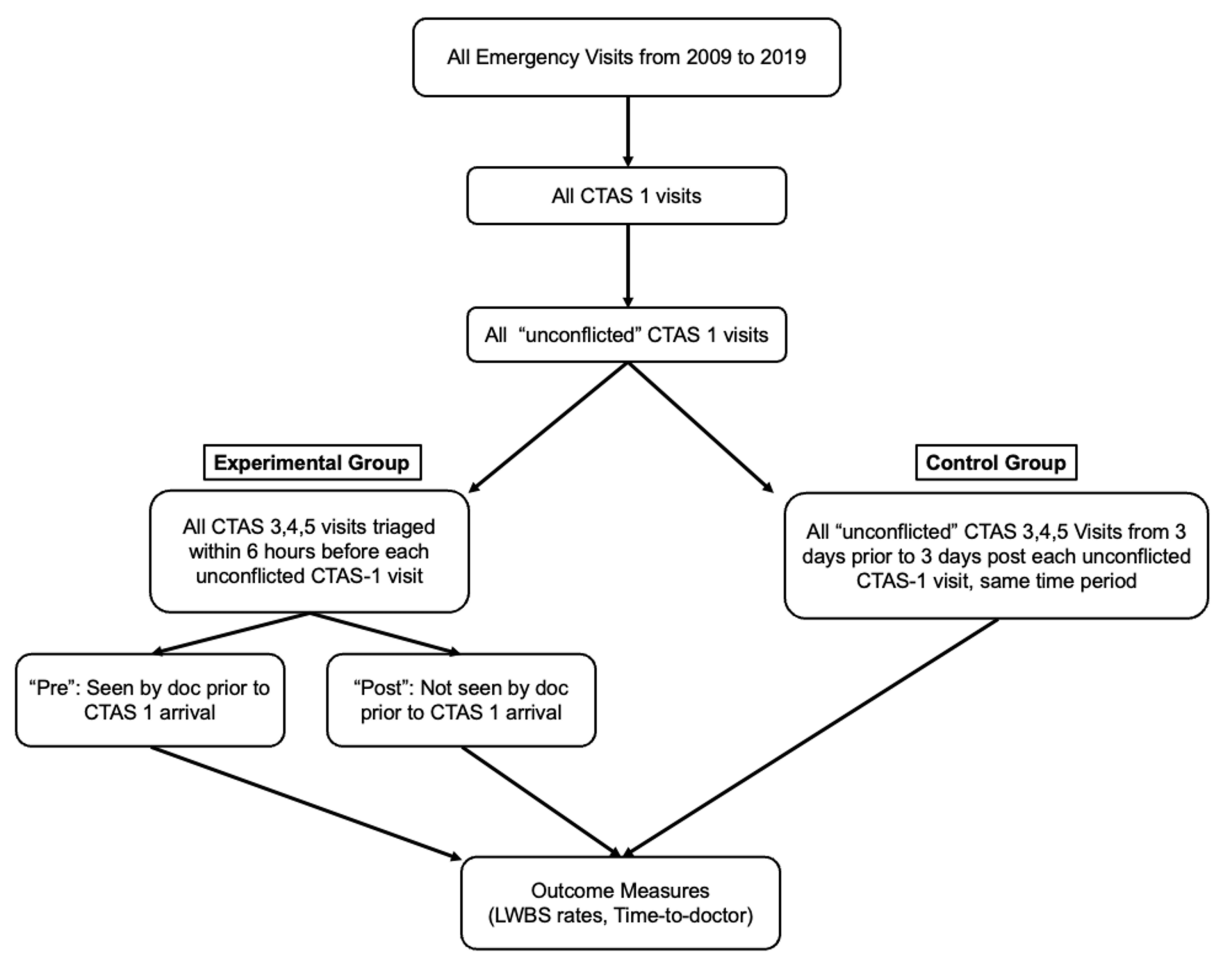
Data acquisition pathway. “Unconflicted” refers to those visits that were at least 12 hours from any previous CTAS 1 visit and at least six hours from any subsequent CTAS 1 visit. We only included data where the CTAS 1 visit was both unconflicted and had unconflicted controls. CTAS: Canadian Triage and Acuity Scale

To-Doctor times for CTAS 3, 4, or 5 visits triaged within six hours of each unconflicted CTAS 1 event were identified. These To-Doctor times were coded as “pre” if the first physician contact had occurred prior to the CTAS 1 event, and “post” if following. To-Doctor times were defined as the time from patient registration to first physician contact. The timestamps used for our analysis reflect that of electronic record entry of patient registration and the time the patient seen as written on the chart by the seeing physician. Figure 1 demonstrates our data acquisition pathway. 

TTA events were defined as presentations involving the activation of our center’s trauma team, as per institutional activation guidelines. Non-TTA CTAS-1 events were defined as cases triaged as CTAS-1, but not fulfilling criteria for a TTA (e.g., cardiorespiratory arrest, shock).

To obtain controls, for each unconflicted CTAS 1 event, visits triaged as CTAS 3 to 5 that occurred within six hours of the same time from each day for three days prior to and three days after the index CTAS 1 event, were reviewed. If these were not CTAS 1 conflicted (as defined above), they were included as controls, labelled “pre” and “post” accordingly. Our final data set included only unconflicted CTAS 1 events that a) had both pre- and post-study data and b) had both pre- and post-control data.

The change in To-Doctor times for each CTAS 3-5 visit for both study and control data was calculated as a percentage of pre-times (Change = (Post-Pre)/Pre). Averages were calculated for study and control tuples and compared using Student's t-tests. To account for potential changes in ED protocols or staffing, our data was divided into temporal quartiles and repeated for each quartile.

Patients who LWBS within six hours of a CTAS-1 event were identified. LWBS rates were compared between TTAs and non-TTA CTAS-1 events.

Survey methods

We distributed an online, anonymous, voluntary questionnaire to a multidisciplinary group of ED staff in the IWK Health Centre Emergency Department in December 2021, including physicians, resident physicians, nurses, ward clerks, unit aides, and protection services. Study data were collected and managed online using Opinio (ObjectPlant Inc., Oslo, Norway). The survey was developed in consultation with PED staff. Face validity was determined by trauma and ED content experts via pilot testing with a small group. Internal consistency of the survey answers was measured by Cronbach’s alpha.

Participants consented to voluntary survey participation through an online form. This survey was reviewed and approved by the (institution omitted) Research Ethics Board (#1027334). The IWK Health Centre PED is a tertiary urban site that treats approximately 36000 patients per year. The site serves as the only Level 1 Pediatric trauma center in the (location omitted for blinding).

At our center, the trauma team consists of a trauma team lead, ED nurses, general surgery, orthopedics, pediatric intensive care unit staff and residents, radiology technologists, laboratory technicians, and respiratory therapists. Other delayed response members include radiology staff/resident, CT technologist, neurosurgery staff/resident, and social work, as needed. In a CTAS-1 resuscitation case, the team consists of an emergency physician, several nurses, respiratory therapy and other allied health providers as needed.

Participants answered 12 Likert scale questions on their perception of the effects of TTAs and non-TTA CTAS-1 resuscitations on PED operations. Questions are presented in Supplemental Appendix A. Quantitative survey responses were summarized using means and differences between groups were calculated using Mann-Whitney U testing. Bonferroni correction was used to adjust for multiple comparisons. Multiple logistic regression was used to adjust for provider experience, gender, and age. In the logistic regression model, Likert scores were converted to binary values, comparing strongly agree/agree responses to strongly disagree, disagree, and neither agree/disagree to compare groups. GraphPad Prism version 10.0 for Macintosh (Boston, USA) was used for statistical analyses. 

Participants were also asked to answer five free-text questions to share their additional thoughts and describe aspects of department flow they feel are affected by TTAs and other CTAS-1 resuscitations (Supplemental Appendix A). We employed a thematic analysis in the exploration of free-text comments. Text was manually coded by two investigators employing consensus coding with key phrases identified from responses. Lastly, participant comments were organized into four themes: staff shortages, disruptions to care, resource utilization, and quality improvement.

## Results

Visit data results

**Figure 2:**
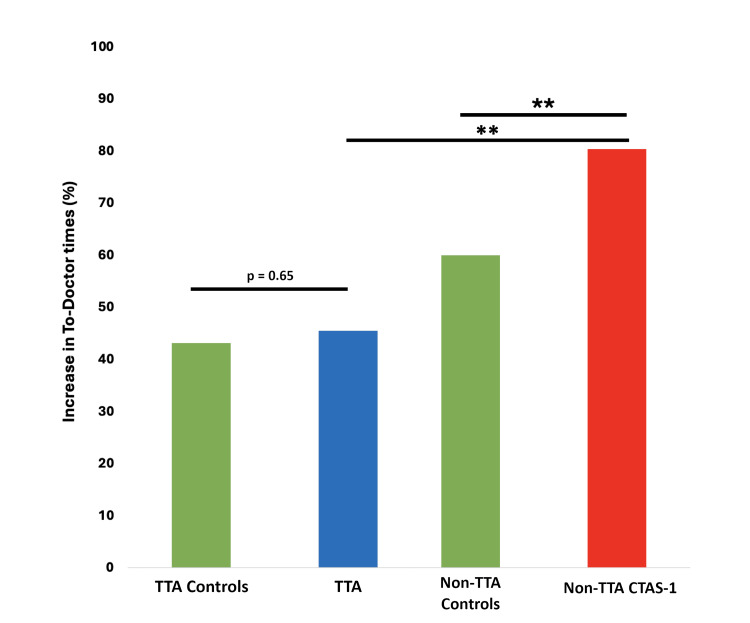
To-doctor times are significantly increased after non-TTA CTAS-1 events. Controls were obtained for index TTA and non-TTA CTAS-1 cases separately. For each unconflicted CTAS 1 event, visits triaged as CTAS 3 to 5 that occurred within six hours of the same time from each day for three days prior and three days after the index CTAS 1 event were reviewed. p-values shown for pairwise Student's t-test comparisons (** = p < 0.001). TTA: Trauma team activation; CTAS: Canadian Triage and Acuity Scale

ED visit data analysis from 2009 to 2019 identified 1305 visits that met study inclusion criteria. To-doctor times for visits labeled as CTAS-3, 4, or 5 were collected for patients triaged within six hours of each CTAS-1 event. TTA events did not significantly increase to-doctor times (Figure 2). For non-TTA CTAS-1 visits, to-doctor times were significantly increased post CTAS-1 event compared to both controls (p<0.001) and TTA visits (p<0.001). Patients who left without being seen did not significantly differ between non-TTA CTAS-1 and TTA events (1.76 patients vs 1.60, p=0.43).

Survey demographics

The survey had a total of 51 respondents of approximately 150 staff who received the survey email (response rate: 34%). Of those who responded, 19 were registered nurses, 20 physicians (14 staff ED physicians, including two trauma team leaders (TTLs), and four residents), seven ward clerks and unit aides, and four held other positions in the ED (including protection services and nurse practitioners). Respondents were 59% female. Physicians were PEM-fellowship trained (n=6), followed by pediatricians (n=4) and FRCPC/CCFP EM trained (n=4). Respondents chose a single role that best reflected their role in the emergency department. The majority of respondents were involved in at least five TTAs (5-10 TTAs, 25.5% of respondents; more than 10 TTAs, 49% of respondents). Table 1 contains a complete summary of demographic information. Cronbach’s alpha for all Likert-scored questions, in all respondents, was 0.94. In the RN group, Cronbach’s alpha was 0.77, and 0.82 in the MD group.

**Table 1:** Study participant demographics.

Role	Number	%
RN	19	37.30%
ED Physician (with a PEM fellowship)	6	11.80%
ED Physician (Pediatrics)	4	7.80%
Ward Clerk	4	7.80%
Protection services	4	7.80%
ED Physician (FRCPC EM)	3	5.90%
Unit Aide	3	5.90%
Resident (FRCPC)	2	3.90%
Physician (trauma team lead)	2	3.90%
Resident (CCFP)	1	2.00%
Resident (PEM fellowship)	1	2.00%
ED Physician (CCFP-EM)	1	2.00%
Nurse practitioner	1	2.00%
Age		
20 - 30	12	23.50%
31 - 40	18	35.30%
41 - 50	15	29.40%
51 - 60	3	5.90%
61 - 70	3	5.90%
Gender		
Female	30	58.80%
Male	21	41.20%
How many trauma team activations (TTAs) have you been involved in during your time in the ED?		
0	3	5.90%
1	3	5.90%
2	1	2.00%
Less than 5	6	11.80%
5 to 10	13	25.50%
More than 10	25	49.00%
How many high acuity (CTAS 1) resuscitations (e.g., sepsis, cardiac arrest, status epilepticus, severe asthma) have you been involved in during your time in the ED?		
0	4	7.80%
1	2	3.90%
2	1	2.00%
Less than 5	8	15.70%
5 to 10	9	17.60%
More than 10	27	52.90%

Perception of TTAs and non-TTA CTAS-1 cases on departmental flow and patient safety

**Figure 3:**
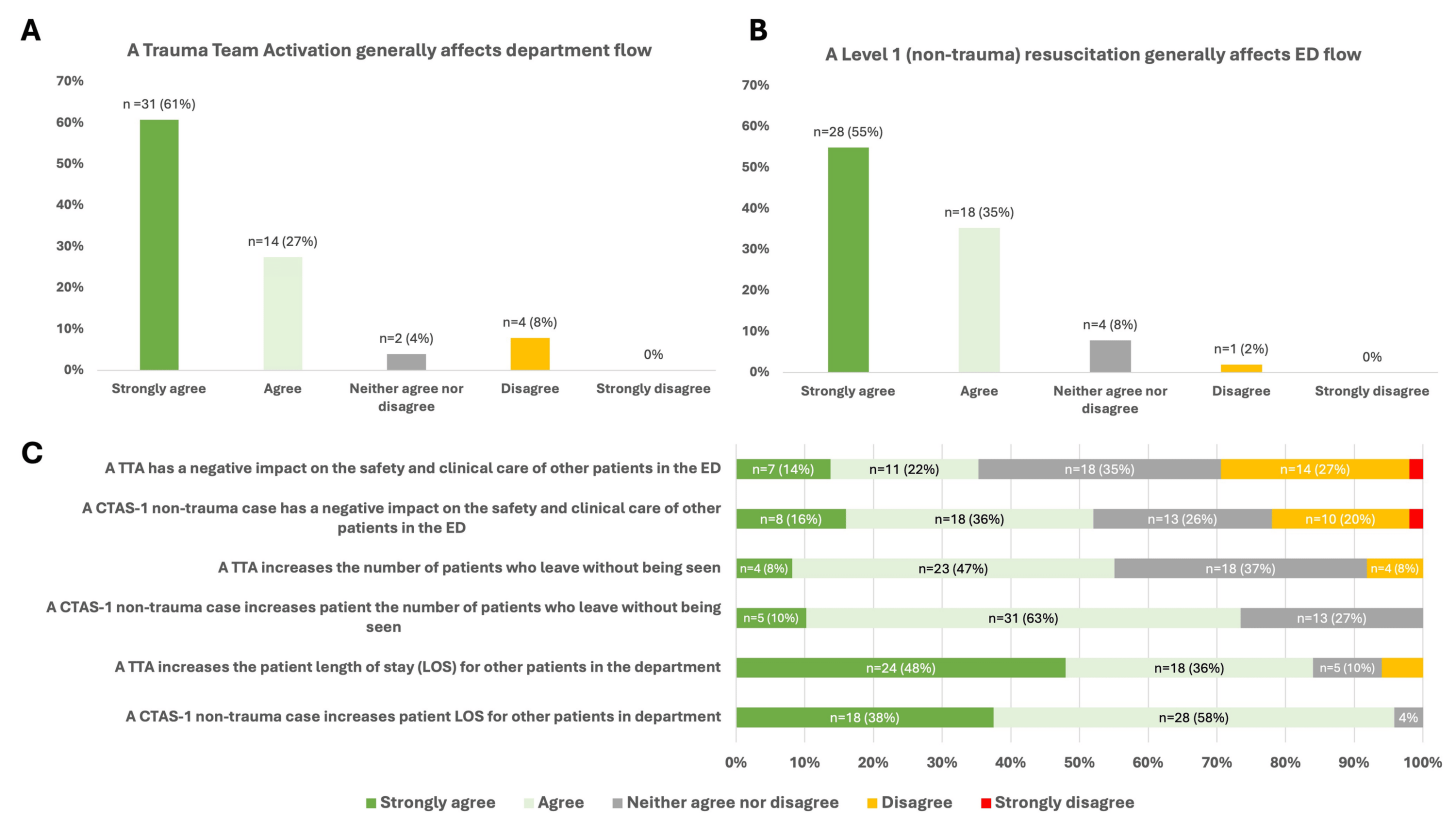
Participant responses (n=51) to questions regarding trauma team activations and non-TTA CTAS-1 cases. The majority of participants (n=51) strongly agreed or agreed that TTAs (A) and non-TTA CTAS-1 cases (B) affect ED flow. Overall, respondents perceived that both trauma team activations (TTAs) and non-TTA CTAS-1 cases increase emergency department length of stay for other patients, while opinions were mixed regarding their impact on safety, clinical care, and rates of patients leaving without being seen (Panel C). CTAS: Canadian Triage and Acuity Scale

Most participants (88% and 90% of respondents, respectively) had the perception that TTAs and non-TTA CTAS-1 cases affect department flow, increase patient LOS, and increase LWBS rates (Figure 3). Ninety percent of participants agreed or strongly agreed that they have a strong sense of teamwork during both TTAs and non-TTA CTAS-1 resuscitations. The majority of respondents (52%) agreed that a non-TTA CTAS-1 case has a negative effect on the safety and clinical care of other patients in the ED, while 36% of respondents agreed on the same for TTAs (Figure 3). Answers to all survey questions are shown in Supplemental Appendix B. 

Inter-provider variability in perception of high-acuity cases on departmental flow

Trauma Team Activations

A greater proportion of nursing staff compared to physicians agreed that a TTA has a negative impact on the safety and clinical care of other patients in the ED, which was not significant after Bonferroni correction (60% vs 15%, p=0.025, pcorr = 0.45) (Table 2). All non-MD/RN staff studied (n=10) agreed or strongly agreed that TTAs affect department flow. A greater proportion of this group, compared to all others studied, agreed that a TTA increases patient LWBS rates, which was insignificant after Bonferroni correction (77.8% vs 53.1%, p=0.014, pcorr =0.25).

**Table 2:** Varying perceptions of CTAS-1 cases by provider role. Mann-Whitney U testing was carried out with Bonferroni-adjusted values shown. TTA: Trauma team activation; CTAS: Canadian Triage and Acuity Scale

Question	Group 1	Group 2	Group 1 %	Group 2 %	Raw p-value	Bonferroni-adjusted p-value
A non-TTA CTAS-1 case has a negative impact on the safety and clinical care of other patients in the ED	Nurses	MD	n=16 (80%)	n=6 (30%)	0.0022	0.036
A TTA has a negative impact on the safety and clinical care of other patients in the ED	Nurses	MD	n=12 (60%)	n=3 (15%)	0.025	0.45
A TTA increases the number of patients who leave without being seen	Unit aides, ward clerks, protection services	RNs + MDs	n=8 (77.8%)	n=21 (53.1%)	0.014	0.25

Non-TTA CTAS-1 resuscitations

When asked if non-TTA CTAS-1 cases have a negative impact on the safety and clinical care of other patients in the ED, a greater proportion of nursing staff agreed compared to physicians (80% vs 30%, p=0.0022, pcorr = 0.036) After adjusting for age, gender, and clinical experience in a multiple logistic regression model, nursing staff had significantly higher adjusted odds of this perception compared to physicians (OR 29.7, p = 0.0038) (Table 2).

No differences were identified based on degree of experience with TTAs or between levels of physician training. In addition, no significant response differences were identified between residents and staff physicians.

Qualitative results

Through thematic analysis, we identified four themes from participants when asked about aspects of ED flow affected by TTAs and non-TTA CTAS-1 cases: staff shortage, disruptions to care, resource utilization, and quality improvement.

Staff shortages

A consistent theme identified in the free-text comments was that of staff shortages during all CTAS-1 cases, primarily regarding competing demands of nursing staff and physician availability during a high-acuity case. Several other respondents reiterated the resource shortage when the TTL is an on-shift emergency physician. It was also stated that at least three nurses are needed to effectively manage a TTA, placing stress on the rest of the department, given our ED is staffed by 5-7 RNs at one time. One respondent noted that ward clerks, X-ray, and blood work technicians all need to attend the TTA, with a nurse occasionally being taken from triage.

Disruptions to care

Several respondents noted disruptions to routine ED care during TTA and non-TTA CTAS-1 cases. Respondents felt that medication administration, reassessments, and diagnostic imaging times are affected. Others believed that TTAs and non-TTA CTAS-1 cases create a nursing shortage for other patients who may need urgent care, frequent reassessments, procedures (e.g., orthopedic reductions), and medication administration. During CTAS-1 cases, one participant noted that communication with patients in the waiting room was “less timely” due to staff being needed at the high-acuity case.

Regarding patient safety, one participant noted that “The staff that remains on the floor are left struggling to keep up with the rest of the patients who are also sick/require frequent reassessments.” One respondent noted that non-TTA CTAS-1 cases may have a greater impact on patient flow: “This typically has a larger impact on patient flow as the Emergency Physician is leading the resuscitation, so is not available to continue care of other patients until this Level 1 patient is stabilized. This also takes nursing, ward clerk, blood collection, and diagnostic imaging support, so those resources are prioritized for the Level 1 patient, and others wait.”

Resource utilization

Comments from staff reiterated that TTAs and non-TTA CTAS-1 cases are highly resource-intensive. Staffing resources are strained, as mentioned above, in addition to access to PPE and other services such as diagnostic imaging. Several participants also noted that if specialists are needed for a high-acuity case, this may delay care for other patients in need.

Quality improvement

We asked respondents to suggest ideas for the development of quality improvement initiatives. Suggestions included assistance from other hospital RN staff for resuscitation, relay of updated information to waiting rooms, and additional simulations. A summary of proposed quality improvement initiatives is presented in Table 3. 

**Table 3:** Suggestions from participants for improving departmental flow during TTAs or CTAS-1 non-trauma cases. TTA: Trauma team activation; CTAS: Canadian Triage and Acuity Scale; TTL: trauma team leader

Suggestions for improving department flow
Have assistance from other hospital nursing staff (e.g. OR/PICU) to assist in resus room
Do not overlap attending ED physician and TTL coverage
Relay information to waiting rooms via updated TV screens
If physicians could take on nursing tasks during busy periods (e.g., NP swabs, postural vitals, urinalysis), if appropriate
Quick “running of the board” if possible by staff – to determine which non-emergent patients can have their disposition determined before the case arrives
Streamlined admission process, involving quicker identification of whether a patient will require admission
Additional interdisciplinary, in-situ, simulations

Aspects of department flow: CTAS-1 (non-trauma) resuscitations

Similar concerns as with TTAs were discussed with non-TTA CTAS-1 resuscitations (e.g., nursing shortages, slower reassessments, staff being occupied). Several participants stated that non-TTA CTAS-1 cases have a larger impact on patient flow compared to TTAs, due to being time- and resource-intensive.

## Discussion

We performed a combined qualitative and quantitative analysis of the effects of TTAs and other high-acuity cases on PED flow and patient care at a single tertiary pediatric trauma center. A notable finding of this study is that TTAs were not associated with statistically significant increases in patient time-to-doctor or LWBS rates. In contrast, non-TTA CTAS-1 events were associated with longer wait times for lower-acuity patients and were perceived by staff as more disruptive to routine department operations. Our findings highlight staff perceptions and the objective data regarding the impact high-acuity cases have on PED flow. Our visit data analysis suggests a longer wait time for patients triaged as CTAS-3, -4, or -5 during a non-TTA CTAS-1 event. This finding aligns with perceptions expressed by survey respondents, particularly from nurses, who reported that non-TTA CTAS-1 cases created greater disruption compared to TTAs. By contrast, TTAs were not associated with a statistically significant increase in to-doctor times, nor were they linked to an increase in rates of patients who LWBS. These differences observed between visit data and provider perceptions of TTAs and non-TTA CTAS-1 cases suggest that the structured nature of TTAs may contribute to reduced disruption in our PED. Importantly, these findings suggest that the disruptive effects of a high-acuity event may depend not only on patient acuity but also on the structure and organization of the response system mobilized to manage it.

Non-TTA CTAS-1 cases are variable in presentation (i.e., cardiac arrest, severe respiratory distress, altered level of consciousness). The approach to non-trauma cases varies, is unpredictable, and is suggested to be more impactful on department crowding based on our survey responses. Staff highlighted concerns about the unpredictable nature of non-trauma resuscitation and the lack of a formal structure compared to TTAs. Our limited analysis of these high-acuity events suggests an association that non-TTA events may impact PED resources more acutely, as multiple nurses, physicians, and support staff are pulled away from routine patient care responsibilities. Trauma team responses are standardized, well-practiced, and engage a defined multidisciplinary team beyond the PED, which may partially offset the resource demands placed on the department [5,6]. Team members typically have clearly delineated roles and responsibilities, which may reduce uncertainty and limit the diversion of resources from ongoing patient care. By comparison, non-trauma CTAS-1 presentations are heterogeneous and may include conditions such as cardiac arrest, severe respiratory distress, or altered level of consciousness. These presentations often require ad hoc team assembly and role allocation, creating greater operational uncertainty. Survey respondents frequently highlighted this lack of structure and predictability as a source of disruption. Analysis of our visit data suggests that these non-TTA CTAS-1 cases may be more disruptive to department flow compared to TTA CTAS-1 patients.

This visit data analysis was reinforced by staff survey results. A greater proportion of nursing staff perceived negative effects from TTAs and non-TTA CTAS-1 cases compared to physicians surveyed. This result was statistically significant after multiple-test correction only with non-TTA CTAS-1 cases and remained significant with logistic regression modelling controlling for age, experience, and gender. Reasons for this variability in perception may be due to RNs having a direct involvement in tasks like medication administration, bloodwork, and other care, which are more likely to be delayed during a high-acuity case. Additional comments from nursing staff specifically stated that communication with waiting room patients is less timely, due to staff who normally interact with these patients being occupied with the high-acuity case. Our survey responses highlight the potential operational impact on the rest of the department during high-acuity cases, such as that on delays in patient triage, medication administration, or delays in low-acuity procedures. Previous work has demonstrated that prolonged patient length of stay in the ED negatively affects RN perception of safe care [13]. Although not externally validated, our survey was internally consistent with high Cronbach’s alpha measures of survey responses.

Strengths and limitations

To our knowledge, this is the first study examining the influence of high-acuity cases on PED flow while incorporating provider perception of CTAS-1 cases. Our findings parallel broader work in the literature on ED crowding and its detrimental effects. For example, crowding has been linked to prolonged length of stay, delays in care, increased LWBS rates, and poorer patient outcomes [11,12]. However, our study contributes unique insight by disentangling the effects of trauma-specific and non-trauma-specific high-acuity cases, and by examining both quantitative metrics and provider perceptions.

Our study is limited by being conducted at a single center, with 51 participants completing the survey. Our response rate was 34% of ED staff, which may introduce participation bias into our study. Given relatively small sub-group sizes, comparisons between RNs and MDs may be underpowered. Replication of this analysis at other sites would be beneficial to glean further information on the effects of high-acuity cases on PED department flow, as this single-center analysis has limited generalizability. Other limitations of this analysis include being unable to correct for other dynamic patient and personnel factors in the PED, such as the acuity level of other patients in the department and sudden changes in staffing. We were also unable to control for other operational metrics including baseline department occupancy, concurrent patient acuity, and specific nursing ratios at the time of the event. Our outcome measures were defined only as patient time-to-doctor and LWBS rates, which do not fully encapsulate department flow and patient outcomes. The controls obtained in our dataset for visit analysis were for visits unconflicted for high-acuity events, at the same time of day (six-hour period), three days before and after the conflicted event. We felt this was an accurate measure of similar visits, but it does not account for other department flow metrics as mentioned above. Our qualitative findings are also subject to response and recall biases, common in self-reported survey data. Future multi-center studies utilizing more granular staffing data and other flow metrics are needed to validate these site-specific observations.

Clinical implications

PED flow is complex, with many factors influencing flow and congestion [14]. These include volume and urgency of patients, triage resources, PED staffing, complexity and acuity of care needs, and overall service efficiency. Several of these factors were highlighted by comments from our survey participants.

Comments obtained from staff may inform quality improvement efforts for management of all CTAS-1 cases and provide insight into how best to structure high-acuity case response teams. Such efforts could enhance overall departmental flow while considering the experience and challenges of staff in the ED. Given our findings suggest raising the possibility that non-TTA CTAS-1 events may impact PED resources to a greater degree, these cases may also benefit from structured response teams, like TTAs. A study of an adult quaternary care facility showed that implementation of an intensivist-led medical emergency team decreased cardiac arrest rates in critically ill ED patients [15].

The findings of this study suggest that the operational advantages of TTAs may stem not only from the resources mobilized but from the structured and standardized nature of the response itself. Utilizing elements of trauma team organization and response to selected non-trauma CTAS-1 presentations may help mitigate their impact on PED flow.

## Conclusions

This single-center study examined PED visit data and staff perceptions of the effects of TTAs and other high-acuity cases on PED flow and patient care. Through surveying a multidisciplinary sample of health care professionals and support staff in a tertiary PED, we identified key perceptions regarding department flow during TTAs and other high-acuity resuscitations. Additionally, our qualitative findings suggest raising the possibility that non-TTA CTAS-1 cases are perceived as more disruptive to department flow compared to TTAs. Although our study is underpowered to find causal mechanisms for these suggested changes, they highlight important potential evidence for the differential operational impact of high acuity in the PED. This study provides new insight into the impact of high-acuity cases on PED flow from the healthcare provider perspective.

## Appendices

Appendix A - survey questions

1. What is your primary role in the emergency department (ED)?

2. What is your age?

3. What gender do you identify with?

4. How many trauma team activations have you been involved in during your time in the IWK ED?

5. How many high acuity (CTAS 1) resuscitations (e.g., sepsis, cardiac arrest, status epilepticus, severe asthma) have you been involved in during your time in the IWK ED?

Rate your level of agreeability with the following statements from 1 (Strongly Disagree) to 5 (Strongly Agree):

6. A Trauma Team Activation generally affects department flow

7. A Trauma Team Activation increases the patient length of stay (LOS) for other patients in the department

8. A Trauma Team Activation increases the number of patients who leave without being seen

9. A Trauma Team Activation has a negative impact on the safety and clinical care of other patients in the ED

10. A Level 1 (non-trauma) Resuscitation generally affects ED flow

11. A Level 1 (non-trauma) Resuscitation increases the patient length of stay (LOS) for other patients in the department

12. A Level 1 (non-trauma) Resuscitation increases the number of patients who leave without being seen

13. A Level 1 (non-trauma) Resuscitation has a negative impact on the safety and clinical care of other patients in the ED

14. I have a strong sense of teamwork when there is a Trauma Team Activation

15. I have a strong sense of teamwork when there is a Level 1 (non-trauma) Resuscitation

From 1 (not at all) to 5 (all the time):

16. Do you feel in general that there are significant differences in how Level 1 resuscitations impact ED flow versus Level 1 Trauma Team Activations?

Free text comments:

17. Are there any other aspects of department flow that you feel are affected by Trauma Team Activations?

18. Are there any other aspects of department flow that you feel are affected by Level 1 (non-trauma) resuscitations?

19. Do you have any suggestions for how we can improve departmental flow when there are Trauma Team Activations?

20. Do you have any suggestions for how we can improve departmental flow when there are Level 1 (non-trauma) resuscitations?

21. Do you have any other comments?

Appendix B

**Table 4:** Summarized survey respondents for all Likert-scale questions. TTA: Trauma team activation

	Strongly Disagree	Disagree	Neither agree nor disagree	Agree	Strongly Agree
A Trauma Team Activation generally affects department flow	0.0%	7.8%	3.9%	27.5%	60.8%
A TTA increases the patient length of stay (LOS) for other patients in the department	0.0%	6.0%	10.0%	36.0%	48.0%
A TTA increases the number of patients who leave without being seen	0.0%	8.2%	36.7%	46.9%	8.2%
A TTA has a negative impact on the safety and clinical care of other patients in the ED	2.0%	27.5%	35.3%	21.6%	13.7%
A Level 1 (non-trauma) resuscitation generally affects ED flow	0.0%	2.0%	7.8%	35.3%	54.9%
A Level 1 (non-trauma) Resuscitation increases the patient length of stay (LOS) for other patients in the department	0.0%	0.0%	4.2%	58.3%	37.5%
A Level 1 (non-trauma) Resuscitation increases the number of patients who leave without being seen	0.0%	0.0%	26.5%	63.3%	10.2%
A Level 1 (non-trauma) Resuscitation has a negative impact on the safety and clinical care of other patients in the ED	2.0%	20.0%	26.0%	36.0%	16.0%
I have a strong sense of teamwork when there is a TTA	0.0%	2.0%	8.2%	36.7%	53.1%
I have a strong sense of teamwork when there is a Level 1 (non-trauma) resuscitation	0.0%	2.0%	8.0%	46.0%	44.0%
	One (not at all)	Two	Three	Four	Five (all the time)
Do you feel in general that there are significant differences in how non-TTA level 1 cases impact ED flow vs Level 1 TTAs?	2.1%	14.6%	35.4%	33.3%	14.6%

## References

[1] Sahai VS, Ward MS, Zmijowskyj T, Rowe BH (2005). Quantifying the iceberg effect for injury: using comprehensive community health data. Can J Public Health.

[2] Billette J-M, Janz T: Injuries in Canada (2025). Insights from the Canadian Community Health Survey. https://www150.statcan.gc.ca/n1/pub/82-624-x/2011001/article/11506-eng.htm.

[3] Petrie D, Lane P, Stewart TC (1996). An evaluation of patient outcomes comparing trauma team activated versus trauma team not activated using TRISS analysis. Trauma and Injury Severity Score. J Trauma.

[4] Noonan M, Olaussen A, Mathew J, Mitra B, Smit V, Fitzgerald M (2019). What is the clinical evidence supporting trauma team training (TTT): a systematic review and meta-analysis. Medicina (Kaunas).

[5] Cameron M, McDermott KM, Campbell L (2019). The performance of trauma team activation criteria at an Australian regional hospital. Injury.

[6] Nonis M, McCombie A, Wakeman C, Fleischer D, Joyce L (2022). Trauma team activation: improved care of major trauma patients. N Z Med J.

[7] Vernon DD, Furnival RA, Hansen KW, Diller EM, Bolte RG, Johnson DG, Dean JM (1999). Effect of a pediatric trauma response team on emergency department treatment time and mortality of pediatric trauma victims. Pediatrics.

[8] Georgiou A, Lockey DJ (2010). The performance and assessment of hospital trauma teams. Scand J Trauma Resusc Emerg Med.

[9] Bullard MJ, Musgrave E, Warren D (2017). Revisions to the Canadian Emergency Department Triage and Acuity Scale (CTAS) Guidelines 2016. CJEM.

[10] Warren DW, Jarvis A, LeBlanc L, Gravel J (2008). Revisions to the Canadian Triage and Acuity Scale paediatric guidelines (PaedCTAS). CJEM.

[11] Bernstein SL, Aronsky D, Duseja R (2009). The effect of emergency department crowding on clinically oriented outcomes. Acad Emerg Med.

[12] Doan Q, Wong H, Meckler G (2019). The impact of pediatric emergency department crowding on patient and health care system outcomes: a multicentre cohort study. CMAJ.

[13] Eriksson J, Gellerstedt L, Hillerås P, Craftman ÅG (2018). Registered nurses' perceptions of safe care in overcrowded emergency departments. J Clin Nurs.

[14] Chan M, Meckler G, Doan Q (2017). Paediatric emergency department overcrowding and adverse patient outcomes. Paediatr Child Health.

[15] Mankidy B, Howard C, Morgan CK (2020). Reduction of in-hospital cardiac arrest with sequential deployment of rapid response team and medical emergency team to the emergency department and acute care wards. PLoS One.

